# Acute administration of NLX-101, a Serotonin 1A receptor agonist, improves auditory temporal processing during development in a mouse model of Fragile X Syndrome

**DOI:** 10.1186/s11689-024-09587-0

**Published:** 2025-01-03

**Authors:** Xin Tao, Katilynne Croom, Adrian Newman-Tancredi, Mark Varney, Khaleel A. Razak

**Affiliations:** 1 Graduate Neuroscience Program, University of California, Riverside, CA USA; 2 Department of Psychology, University of California, 900 University Avenue, Riverside, CA 92521 USA; 3 Neurolixis SAS, Castres, France

**Keywords:** Autism spectrum disorders, Fragile X syndrome, Speech processing, Temporal processing, Sensory hypersensitivity, Serotonin, 5-HT_1A_ receptors

## Abstract

**Background:**

Fragile X syndrome (FXS) is a leading known genetic cause of intellectual disability and autism spectrum disorders (ASD)-associated behaviors. A consistent and debilitating phenotype of FXS is auditory hypersensitivity that may lead to delayed language and high anxiety. Consistent with findings in FXS human studies, the mouse model of FXS, the *Fmr1* knock out (KO) mouse, shows auditory hypersensitivity and temporal processing deficits. In electroencephalograph (EEG) recordings from humans and mice, these deficits manifest as increased N1 amplitudes in event-related potentials (ERP), increased gamma band single trial power (STP) and reduced phase locking to rapid temporal modulations of sound. In our previous study, we found that administration of the selective serotonin-1 A (5-HT_1A_)receptor biased agonist, NLX-101, protected *Fmr1* KO mice from auditory hypersensitivity-associated seizures. Here we tested the hypothesis that NLX-101 will normalize EEG phenotypes in developing *Fmr1* KO mice.

**Methods:**

To test this hypothesis, we examined the effect of NLX-101 on EEG phenotypes in male and female wildtype (WT) and *Fmr1* KO mice. Using epidural electrodes, we recorded auditory event related potentials (ERP) and auditory temporal processing with a gap-in-noise auditory steady state response (ASSR) paradigm at two ages, postnatal (P) 21 and 30 days, from both auditory and frontal cortices of awake, freely moving mice, following NLX-101 (at 1.8 mg/kg i.p.) or saline administration.

**Results:**

Saline-injected *Fmr1* KO mice showed increased N1 amplitudes, increased STP and reduced phase locking to auditory gap-in-noise stimuli versus wild-type mice, reproducing previously published EEG phenotypes. An acute injection of NLX-101 did not alter ERP amplitudes at either P21 or P30, but significantly reduces STP at P30. Inter-trial phase clustering was significantly increased in both age groups with NLX-101, indicating improved temporal processing. The differential effects of serotonin modulation on ERP, background power and temporal processing suggest different developmental mechanisms leading to these phenotypes.

**Conclusions:**

These results suggest that NLX-101 could constitute a promising treatment option for targeting post-synaptic 5-HT_1A_ receptors to improve auditory temporal processing, which in turn may improve speech and language function in FXS.

**Supplementary Information:**

The online version contains supplementary material available at 10.1186/s11689-024-09587-0.

## Background/Introduction

Fragile X syndrome (FXS) is caused by the lack of fragile X messenger ribonucleoprotein (FMRP) and affects approximately 1 in 4000 males and 1 in 8000 females [1]. FXS is the leading known genetic cause of intellectual disability and autism spectrum disorder (ASD)-like behaviors. Fragile X Syndrome (FXS) occurs when the number of CGG repeats in the promoter region of the Fragile X messenger ribonucleoprotein (*Fmr1*) gene exceeds approximately 200. This leads to the gene being transcriptionally silenced, resulting in the loss of the fragile X messenger ribonucleoprotein (FMRP) [2]. Children with FXS show cognitive deficits, repetitive behaviors, anxiety, hyperactivity, seizure susceptibility and sensory hypersensitivity [3–5]. Strong and consistent auditory hypersensitivity impairs daily functioning and may lead to delayed language, high anxiety, and social impairments in FXS. Currently, there are no effective treatments to reduce sensory hypersensitivity in FXS, or other forms of ASD.

Humans with FXS consistently exhibit various sensory processing differences including tactile, visual and auditory hypersensitivity [Tactile: [6]. Visual: [6–8]. Auditory: [8–12]]. The *Fmr1* KO mouse shows many of the sensory phenotypes seen in humans [Tactile: [13], Visual: [7], Auditory: [14], reviewed in [15]], making it a useful animal model for FXS research, particularly for sensory processing abnormalities. An extreme manifestation of auditory hypersensitivity in the *Fmr1* KO mice is audiogenic seizures (AGS), one kind of generalized convulsive seizures induced by loud sounds [16].

Previous studies have shown that activation of serotonin receptors is beneficial in reducing seizures in various epileptic models, including FXS [17–20]. Specifically, FPT ((S)-5-(2’-fluorophenyl)-N, N-dimethyl-1,2,3,4-tetrahydronaphthalen-2-amine), a partial agonist of serotonin-1 A (5-HT_1A_) receptor reduced AGS incidence in *Fmr1* KO mice [17, 21]. Given that FPT is a partial agonist for 5-HT_1A_, 5-HT_2C_ and 5-HT_7_ receptors, the receptor mechanisms underlying its effect on AGS are unclear. A highly selective agonist of the 5-HT_1A_ receptor, NLX-101, attenuated AGS-induced tonic-clonic seizures and death [22]. NLX-101 (also known as F15599) has a higher selectivity for 5-HT_1A_ receptors than the commonly used agonist, 8-OH-DPAT, it preferentially acts on post-synaptic receptors and has minimal effect on somatodendritic auto-receptors in raphe nuclei [23–25], allowing us to narrow down the brain regions and 5-HT_1A_ receptor subpopulations involved.

Electroencephalograph (EEG) recordings have identified remarkably similar auditory processing phenotypes in humans with FXS and the *Fmr1* KO mouse. Physiological measures of auditory hypersensitivity have been observed in humans with FXS, including augmented N1 (first negative peak in sound evoked event related potentials, ERP) amplitudes [8], decreased N1 suppression for repeated sound presentation [9, 12], reduced phase-locking to temporally modulated sound [26] and increased single trial power (STP) [26]. STP is a measure of background power during stimulus processing, and elevated noise may impact temporal processing. Together, these data indicate elevated background and sound induced power, reduced habituation to repeated stimuli and abnormal temporal processing. Such an abnormal cortical milieu is likely to affect normal auditory processing which is required for speech recognition and language function during development. The *Fmr1* KO mice also show robust N1 amplitude elevation [27–29], reduced habituation to repeated stimuli, reduced phase-locking to temporally modulated sound [27, 30] and increased STP [31]. The similarities in EEG phenotypes between humans with FXS and animal models make EEG recordings a promising translational method for evaluation of potential treatments [32]. These recordings provide data supporting target engagement and offer an early indication of efficacy in clinical studies.

A recent study showed juvenile *Fmr1* KO mice had lower whole-brain 5-HT_1A_ receptor expression than WT mice [33], but it remains unclear if agonists of this receptor normalize auditory processing measures in *Fmr1* KO mice. Considering the promising effects of NLX-101 in reducing severity of behavioral auditory hypersensitivity during development [22], we tested the hypothesis that NLX-101 would also reduce EEG measures of auditory hypersensitivity and improve temporal processing. EEG recordings were obtained from *Fmr1* FVB WT and KO mice at two different ages (P21 and P30) with measurements of sound evoked (ERPs) and background responses (non-phase locked STP). The 40 Hz auditory steady state response (ASSR) has been used to study auditory temporal processing and is suggested as a biomarker in neurodevelopmental disorders [34, 35]. Therefore, we used this paradigm to quantify temporal processing.

## Methods

### Mice

All procedures were approved by the Institutional Animal Care and Use Committee at the University of California, Riverside. Mice were obtained from an in-house breeding colony that originated from Jackson Laboratory (Bar Harbor, ME). The mice used for the study are sighted FVB wild-type (Jax, stock# 004828; WT) and sighted FVB *Fmr1* knock-out (Jax, stock# 004624; *Fmr1* KO). The choice of FVB background strain (as opposed to the C57bl6/J strain) for the WT and *Fmr1* KO mice was guided by developmental deficits seen in single unit electrophysiology from auditory cortex [36] and inferior colliculus [37] in FVB mice, as well as temporal processing abnormalities [27]. In addition, NLX-101 reduces audiogenic seizures in this strain [22]. Mice were housed under a 12:12-h light-dark cycle and fed ad libitum. The age ranges and sample sizes used in this study (both males and females) are listed in Table 1. Each mouse was recorded from only once.

**Table 1:**
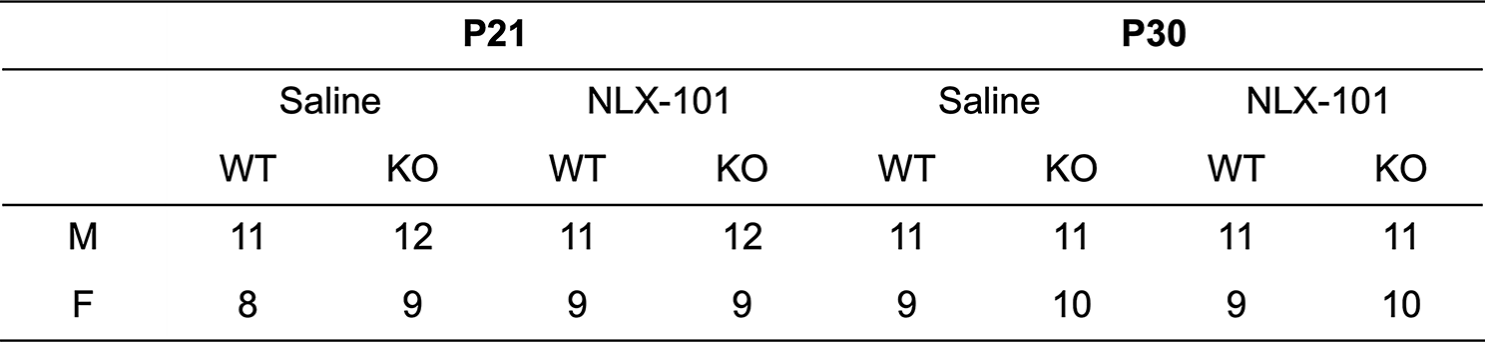
Sample size of the study

### Surgery

Different groups of mice underwent epidural electrode implant surgery at two ages: postnatal (P)18–20 and P27-P29. Mice were anesthetized using intraperitoneal (i.p.) injections of 80/20 mg/kg of ketamine/xylazine. Ketamine supplements were given if necessary. ETHIQA-XR (1-shot buprenorphine, 3.25 mg/kg body weight) was administered via subcutaneous injection prior to surgery as an analgesic. Following the removal of fur (Nair), and sterilization (alcohol and iodine wipes) of the scalp, an incision was made to expose the scalp. A Foredom dental drill was used to drill a hole in the right auditory cortex (at 1.8 mm caudal to bregma and 4.5 mm lateral) as guided by landmarks identified using single unit [14, 36] epidural EEG [29], and depth ERP [38] recordings in the FVB mice. A second hole was made in the right frontal cortex just lateral to the sagittal suture and caudal to the frontal sinus. A third hole, over the left occipital cortex, which served to implant the reference and ground screw electrode, was made in the parietal bone, lateral to the intersection of the sagittal and lambdoid sutures. Three channel electrode posts (Plastics One, MS333-2 A-SPC) were attached to 1-mm stainless steel screws (Plastics One, 8L003905201F) and the screws were advanced into the three pre-drilled holes. Dental cement was applied around the screws, on the base of the post, and exposed skull, to secure the implant. Mice were placed on a heating pad until fully awake and were allowed 48–72 h for recovery before EEG recordings were made.

### Drug administration

NLX-101 (also known as F-15599 - (3-Chloro-4-fluorophenyl-(4-fluoro-4- {[(5-methylpyri- midin-2-ylmethyl)-amino]-methyl}-piperidin-1-yl)-methane-one) was provided as a gift from Neurolixis, Inc. The drug was dissolved in sterile physiological saline and diluted to a dose of 1.8 mg/kg, a dose that reduced AGS in our previous study [22]. In mice, brain concentration of NLX-101 peaks within first 30 min and declines to half concentration after 1 h following intraperitoneal (i.p.) administration (Neurolixis Inc., data on file). Saline or 1.8 mg/kg NLX-101 was given to mice through i.p. injection immediately before EEG recordings. The total duration of the EEG recording was 58 min, including 8 min of resting recording (no stimuli), followed by 30 min of gap-in-noise ASSR and 20 min of broadband noise. The latter two stimuli were counterbalanced in presentation sequence across mice.

### EEG recordings

All EEG recordings were obtained from awake and freely moving mice. EEG recordings were performed at two developmental time points: P20-23 and P29-31, which we refer to as P21 and P30, respectively. Recordings were obtained from the auditory and frontal cortex (AC, FC) electrodes, using the occipital screw as reference. Mice were placed in an arena where they could move freely during the recording. The arena was inside a Faraday cage placed on a vibration isolation table in a sound-insulated and anechoic booth (Gretch-Ken, OR). Mice were attached to an EEG cable via the implanted post under brief anesthesia with isoflurane. The EEG recording set-up has been previously reported [39, 40]. Briefly, the attached cable was connected via a commutator to a TDT (Tucker Davis Technologies, FL) RA4LI/RA4PA headstage/pre-amp, which was connected to a TDT RZ6 multi-I/O processor. OpenEx (TDT) was used to simultaneously record EEG signals and operate the LED light used to synchronize the video and waveform data. Transistor-transistor logic (TTL) pulses were utilized to mark stimulus onsets on a separate channel in the collected EEG data. The EEG signals were recorded at a sampling rate of 24.414 kHz and down-sampled to 1024 Hz for analysis. All raw EEG recordings were visually examined prior to analysis for artifacts, including loss of signal or signs of clipping, but none were seen. Therefore, no EEG data was rejected. Sound evoked EEGs were recorded as follows:

#### Auditory ERP

Broadband noise stimuli (1–12 kHz) were presented at 75 dB SPL (120 repetitions, 100 ms duration, 5ms rise/fall time, 0.25 Hz repetition rate) using a speaker (MF1, Tucker Davis Technologies, FL) situated 20 cm above the floor of the arena. ERP analysis and statistics have been previously described [39, 40]. Briefly, the EEG trace was split into trials, using the TTL pulses to mark sound onset. Each trial was baseline corrected, such that the mean of the 250 ms baseline period prior to sound onset was subtracted from the trial trace for each trial. Each trial was then detrended (MATLAB detrend function) and all trials were averaged together.

To calculate the single trial power (STP) during acoustic stimulation, a time–frequency analysis was performed with a dynamic complex Morlet wavelet transform with Gabor normalization. The wavelet parameter was set for each frequency to optimize time–frequency resolution. Specifically, the wavelet cycles increase as a sigmoidal function from 3 cycles at 1 Hz to a theoretical asymptote of 29 cycles, with an inflection point at 70 Hz and scaling factor of 0.05. The single trial power (STP) does not normalize for baseline power, allowing for the identification of summed stimulus generated and background activity during acoustic stimulation. To compare the responses across genotype at each developmental time point, a non-parametric permutation test was used, to find clusters of significant values [41]. First, a t-test was run on each time-frequency point for the two groups being compared, yielding the T-values for all points. T-values corresponding to *p* < 0.025 were considered significant. Clusters of significant T-values were found, and their area was measured. Next, the group assignments were shuffled randomly, and the t-tests and cluster-measurements were run again on the surrogate groups. This surrogate analysis was performed 2000 times to generate a distribution of cluster sizes that we would expect to find by chance. Originally identified clusters that were larger than 95% of the surrogate clusters were considered significant. This method allows for the identification of significant differences between groups without performing excessive comparisons.

#### Gap-ASSR

The stimulus used to assess auditory temporal processing is termed the ‘40 Hz gap-in-noise ASSR’ (auditory steady state response, henceforth, ‘gap-ASSR’) [40]. The stimulus contains alternating 250 ms segments of noise and gap interrupted noise presented at 75 dB SPL. The gaps were strategically placed 25 ms apart, resulting in a presentation rate of 40 Hz, a rate that produces the strongest ASSR signal when measured from the AC and frontal regions and may reflect the resonance frequency of the underlying neural circuits [42–47]. For each gap-in-noise segment, the gap widths were chosen at random. Gaps of 3–9 ms widths (with 1 ms as a step) and modulation depth of 75% were used. Modulation depth of 75% means the background noise was reduced by 75% during the gaps. To measure the ability of the cortex to consistently respond to the gaps in noise, inter-trial phase clustering (ITPC) at 40 Hz was measured [48]. The ITPC is based on the distribution of phase angles in the EEG response at 40 Hz (because the stimulus is a 40 Hz train) across all trials and reflects the precise timing of 40 Hz activity in the underlying neural generators. The phase angle in EEG recordings refers to the specific position of a response wave cycle at a given point in time. It is a measure of the timing relationship between different brain waves or between brain waves and an external stimuli. The ITPC as defined here quantifies consistency of brain activity as indexed by phase angles in relation to the stimulus across trials. ITPC can be interpreted independently of power. ITPC ranges between 0 and 1, with 0 indicating high variability (uniform distribution) of phase angles across trials, and 1 indicating the same phase angle for every trial. Because ITPC is sensitive to temporal jitter of responses from one trial to the next, this is a useful and commonly used measure of temporal reliability of responses [40, 49–53]. The EEG trace was transformed using a dynamic complex Morlet wavelet transform. The ITPC was calculated for each time-frequency point as the average vector for each of the phase unit vectors recorded across trials (trial count > 100 trials per parametric pair). The ITPC values at 40 Hz were averaged to extract the mean ITPC for the parametric pairs in the AC and FC.

### Statistics

Statistics were performed on GraphPad Prism 9. To evaluate the effects of genotype (2 levels) and treatment (2 levels), multiple Mann-Whitney tests were used for ERP analysis. P-values were adjusted for multiple comparisons with Holm-Sidak method. A three-way ANOVA with repeated measures was used for gap-ASSR analysis, with the three factors being genotype (2 levels), treatment (2 levels), and gap widths. A repeated measures ANOVA was chosen as multiple gap duration data points were collected from a single mouse in a recording session. Greenhouse-Geisser corrections were applied as needed. Post hoc contrasts with Sidak corrections for multiple comparisons were used. Cortical regions (AC, FC) and ages (P21 and P30) were analyzed separately. Male and female data were combined for the main analysis. The supplemental analysis shows effect of sex on measurements, examined by repeated two-way ANOVA with sex and gap width as main factors. Effects of *p* < 0.05 were considered statistically significant, and denoted as *p < 0.05, **p < 0.01, ***p < 0.001, ****p < 0.0001.

## Results

### Larger ERP amplitudes seen in *Fmr1* KO mice were not affected by NLX-101 at P21 or P30

**Fig. 1:**
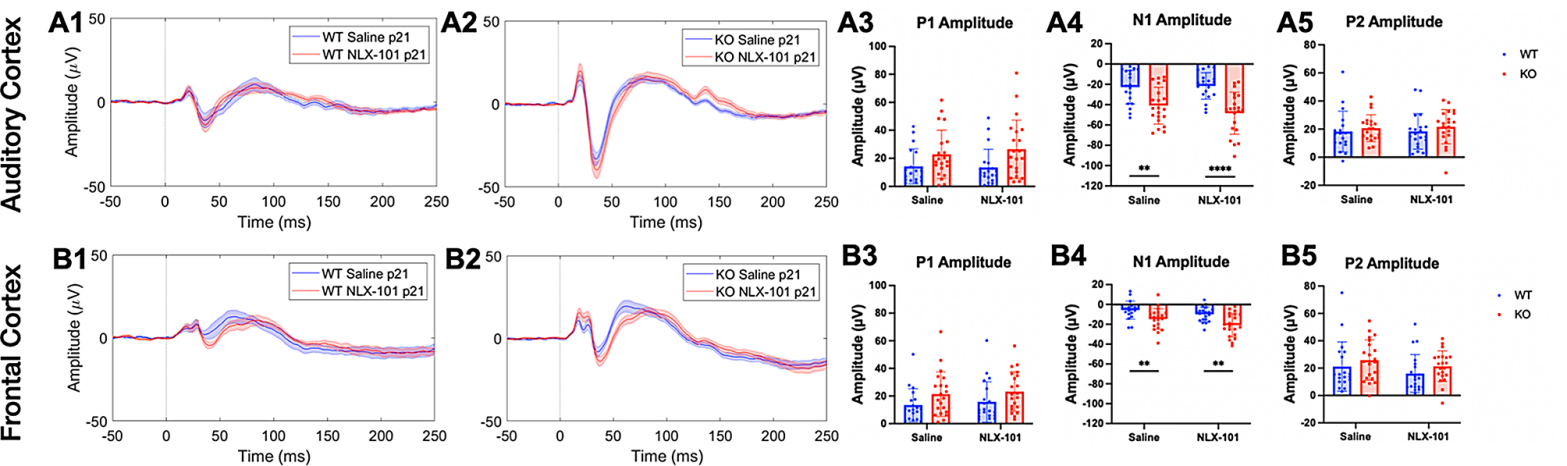
Larger ERP N1 amplitudes in *Fmr1* KO mice at P21 are not corrected by NLX-101. **A1-A5**) ERP in response to noise stimulus recorded from the auditory cortex at P21. **A1-A2**) Grand averaged ERP traces from WT (**A1**) and KO (**A2**) mice, showing treatment comparison. **A3-A5**) Genotype and treatment comparison in P1, N1 and P2 amplitudes. **B1-B5**) ERP responses from the frontal cortex at P21. **B1-B2**) Grand averaged ERP traces from WT (**B1**) and KO (**B2**), showing treatment comparison. **B3-B5**) Genotype and treatment comparison in P1, N1 and P2 amplitudes. In both cortical regions, N1 amplitudes were larger in *Fmr1* KO mice, compared to WT mice regardless of treatment. There were no significant differences in P1 or P2 amplitudes. Full statistics report is in Table 2. *p < 0.05, **p < 0.01, ****p < 0.0001. Error bars show standard deviation

As seen in humans with FXS [7–12], *Fmr1* KO mice show increased amplitude of ERP peaks relative to WT mice [27, 29]. Here we tested whether acute administration of NLX-101 altered auditory hypersensitivity as measured using ERP amplitudes (Fig. 1). At P21, Fig. 1A1-A2 shows average ERP waveforms in the AC in WT and *Fmr1* KO mice, comparing saline and NLX-101. A visual examination of the group average ERP plots from saline treated WT and saline treated KO suggest a genotype difference (compare Fig. 1A1 vs. A2). This is also seen in the FC (compare Fig. 1B1 vs. B2). Significantly increased N1 amplitudes were seen in both AC (Fig. 1A4) and FC (Fig. 1B4) in *Fmr1* KO mice compared to WT mice regardless of treatment (AC: Saline – Multiple Mann-Whitney test. Adjusted *p* = 0.002479 and NLX-101 – Multiple Mann-Whitney test. Adjusted *p* = 0.000041; FC: Saline - Multiple Mann-Whitney test. Adjusted *p* = 0.003659 and NLX-101 - Multiple Mann-Whitney test. Adjusted *p* = 0.003659). At P21, no significant differences were seen in P1 or P2 amplitudes in both AC (Fig. 1A3, A5) and FC (Fig. 1B3, B5). Full statistical results of Fig. 1 are shown in Table 2.

**Table 2:**
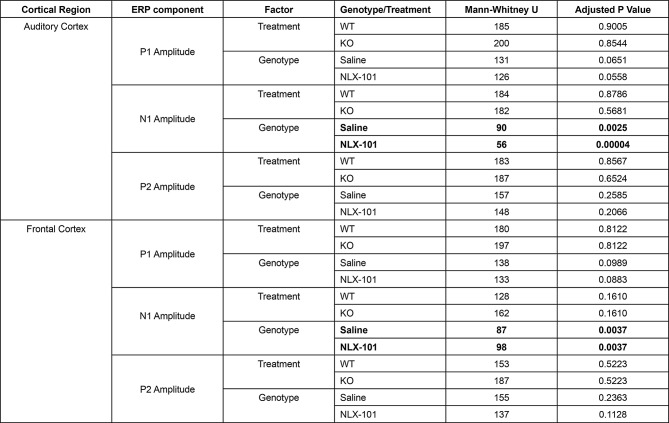
Full statistical analysis of ERP data at P21

**Fig. 2:**
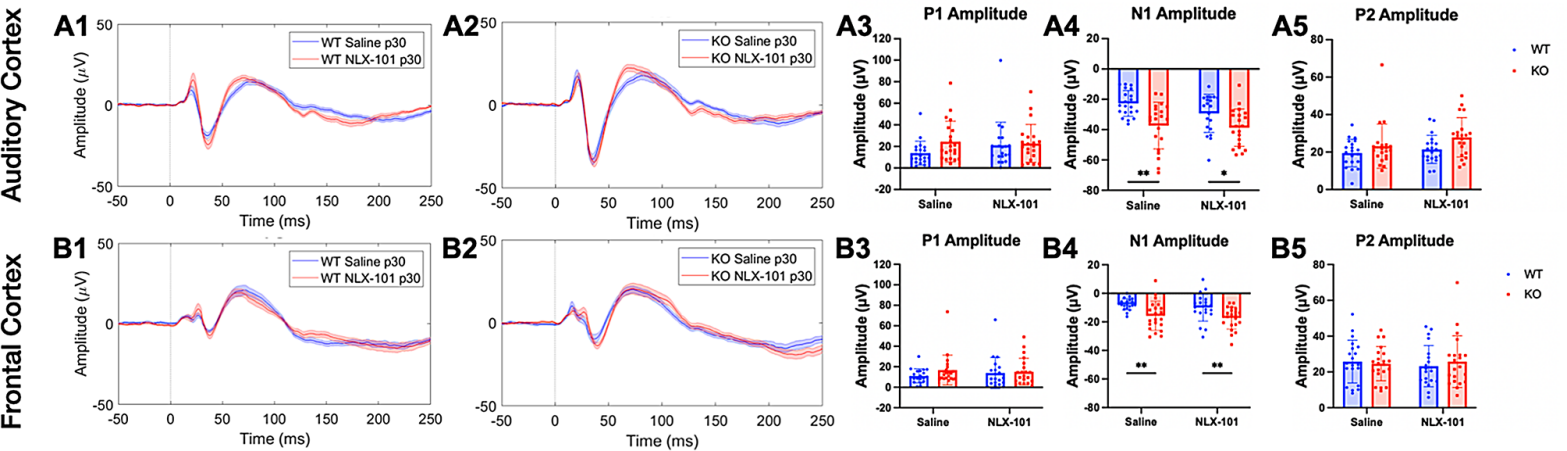
Larger ERP N1 amplitudes in *Fmr1* KO mice at P30 are not corrected by NLX-101. **A1-A5**) ERP in response to noise stimulus recorded from the auditory cortex at P30. **A1-A2**) Grand averaged ERP traces from WT (**A1**) and KO (**A2**) mice, showing treatment comparison. **A3-A5**) Genotype and treatment comparison in P1, N1 and P2 amplitudes. **B1-B5**) ERP responses from the frontal cortex at P30. **B1-B2**) Grand averaged ERP traces from WT (**B1**) and KO (**B2**), showing treatment comparison. **B3-B5**) Genotype and treatment comparison in P1, N1 and P2 amplitudes. In both cortical regions, N1 amplitudes were larger in *Fmr1* KO mice, compared to WT mice regardless of treatment. There were no significant differences in P1 or P2 amplitudes. Full statistics report is in Table 3. *p < 0.05, **p < 0.01. Error bars show standard deviation

Similar results were observed at P30 (Fig. 2). Larger N1, but not P1 or P2, amplitudes were seen in both AC (Fig. 2A3-A5) and FC (Fig. 2B3-B5) in *Fmr1* KO mice compared to WT mice. NLX-101 treatment failed to change N1 amplitude in AC (N1 amplitude: Saline - Multiple Mann-Whitney test. Adjusted *p* = 0.002681 and NLX-101 - Multiple Mann-Whitney test. Adjusted *p* = 0.018409) and FC (N1 amplitude: Saline - Multiple Mann-Whitney test. Adjusted *p* = 0.003279 and NLX-101 -Multiple Mann-Whitney test. Adjusted *p* = 0.004622). Full statistical results of Fig. 2 are listed in Table 3. Taken together, these data indicate that at P21 and P30, N1 amplitude is elevated in the KO mice, but acute NLX-101 treatment does not correct this phenotype.

**Table 3:**
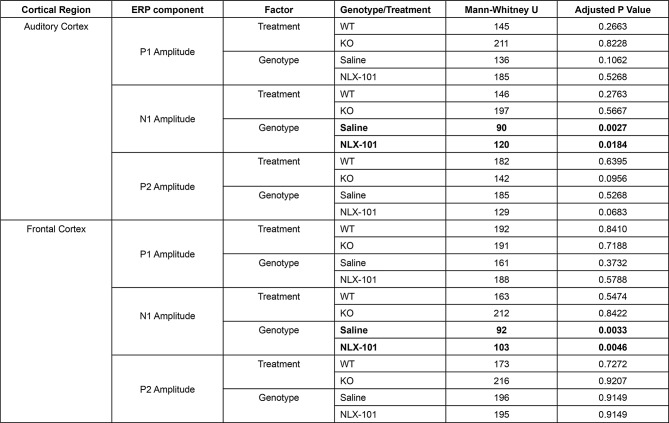
Full statistical analysis of ERP data at P30

### Elevated gamma band single trial power observed in *Fmr1* KO mice was corrected by NLX-101 at P30, but not P21

**Fig. 3:**
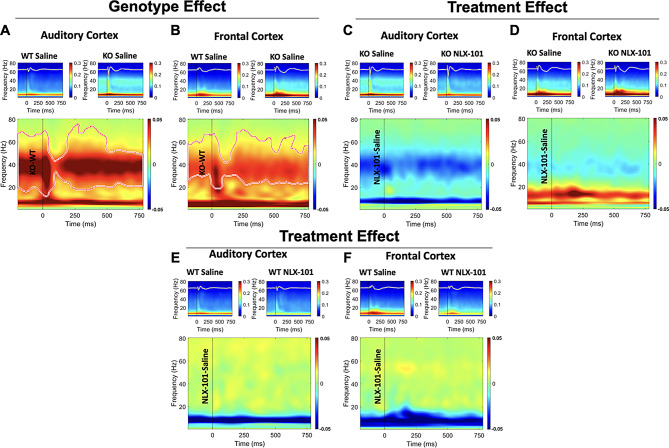
STP is significantly elevated in *Fmr1* KO mice compared with WT at P21, and NLX-101 failed to correct this phenotype. In **A** through **F**, the two smaller panels at the top show grand averaged ERP (as traces) and STP (as heatmaps) from each group. The larger panel at the bottom shows the STP difference between the two groups of mice. The vertical dashed line shows sound onset. The contoured area in the larger panels show regions of significant differences between the group being compared. Warm colors show elevated STP, and cool colors show a reduction in the difference plots. **A-B**) Comparison of saline treated KO and WT mice shows a significant genotype effect on STP at P21. *Fmr1* KO mice have elevated STP in both auditory and frontal cortex compared with WT mice. **C-D**) Comparison of NLX-101 and saline treated *Fmr1* KO mice shows there was no treatment effect in either cortical region at P21. **E-F**) No treatment effect of NLX-101 was seen in WT mice either. The measurement unit for STP is in µV^2^/Hz

A consistent phenotype in both humans with FXS and the *Fmr1* KO mice is elevated single trial power (STP) measured during acoustic stimulation (Human: [9, 54]. Mouse: [30, 31, 55–57]. STP is a measure of background noise at different spectral bands and increased STP may disrupt temporal consistency in auditory responses across trials. We tested if acute NLX-101 reduces STP in *Fmr1* KO mice at P21 and P30. Figure 3A-B shows the genotype effect in saline-treated mice at P21 wherein a significant elevation of STP was concentrated in the gamma spectral band. No significant differences were seen in lower frequency bands. In the AC (Fig. 3A) and in the FC (Fig. 3B), significantly higher gamma STP during ERP measurements was found in saline treated *Fmr1* KO mice compared to WT mice. STP was increased across a broad range of gamma frequencies centered around 40–50 Hz in the KO mice (Fig. 3A-B). At P21, treatment with NLX-101 did not affect *Fmr1* KO (Fig. 3C-D) or WT mice (Fig. 3E-F) compared to saline in either cortical region.

**Fig. 4:**
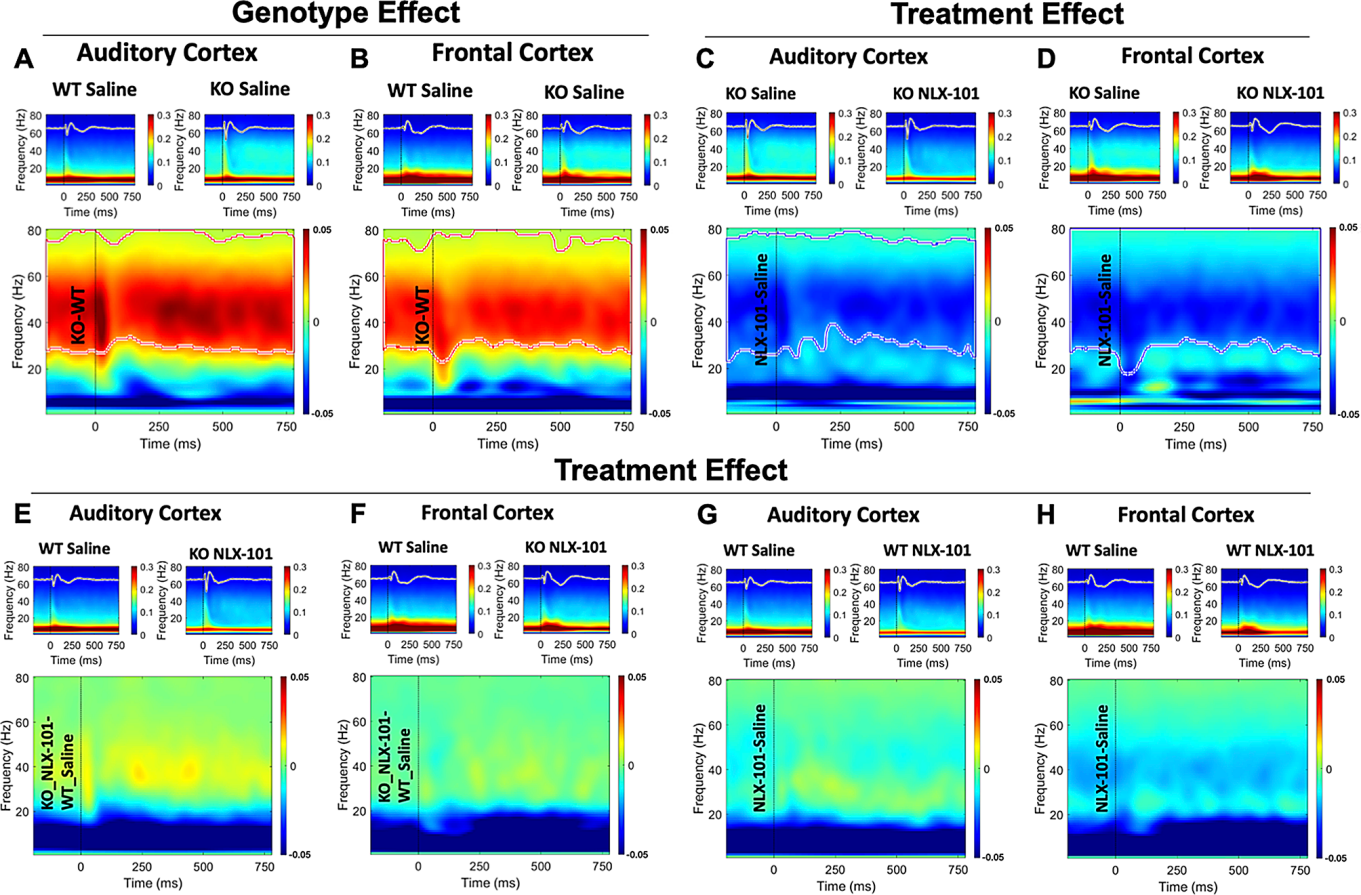
STP is significantly higher in *Fmr1* KO mice compared with WT at P30, and NLX-101 reduced elevated STP in *Fmr1* KO mice without affecting WT mice. The details of this figure are similar to those described in Fig. 3. **A-B**) Comparison of saline treated KO and WT mice shows a significant genotype effect on STP at P30. *Fmr1* KO mice have elevated STP in both auditory and frontal cortex compared with WT mice. **C-D**) STP in *Fmr1* KO mice was significantly reduced after NLX-101 compared with saline treated KO mice. **E-F**) Comparison between KO NLX-101 treated group and WT saline treated group. No difference was seen, suggesting NLX-101 reduced elevated STP in *Fmr1* KO mice to the level that is indistinguishable from WT mice. **G-H**) No treatment effect of NLX-101 was seen in WT mice in either cortical region suggesting NLX-101 effects on STP are specific to *Fmr1* KO mice. The measurement unit for STP is in µV^2^/Hz

At P30, however, NLX-101 reduced gamma band STP in the KO mice (Fig. 4). In saline treated P30 mice, elevated STP was found in *Fmr1* KO mice in both AC (Fig. 4A) and FC (Fig. 4B) compared to WT mice. The elevated STP was only in the gamma frequency band, as seen in P21 mice. Acute NLX-101 administration significantly reduced gamma STP in *Fmr1* KO mice compared to saline in both cortical regions (Fig. 4C-D). There was no significant effect on lower frequency bands. Comparison of NLX-101 treated KO mice to saline treated WT mice showed no difference in STP in both AC (Fig. 4E) and FC (Fig. 4F). NLX-101 did not affect STP in WT groups (Fig. 4G-H). Taken together, these data show significantly increased gamma band STP in both AC and FC at both P21 and P30. NLX-101 had no effect at P21, but significantly reduced this sensory phenotype at P30 to WT levels, suggesting this effect was specific to the *Fmr1* KO mice.

### NLX-101 improves temporal processing at P30

The 40 Hz gap-ASSR is a paradigm in which EEG signals can be recorded in response to gaps presented at 40 Hz in noise. By varying the gap width, the fidelity with which the underlying neural generators phase lock to the temporal modulation across trials can be quantified as the inter-trial phase clustering (ITPC). Our previous study showed cortical deficits in ITPC in developing *Fmr1* KO mice [27]. Here we tested whether NLX-101 improves ITPC in the KO mice.

**Fig. 5:**
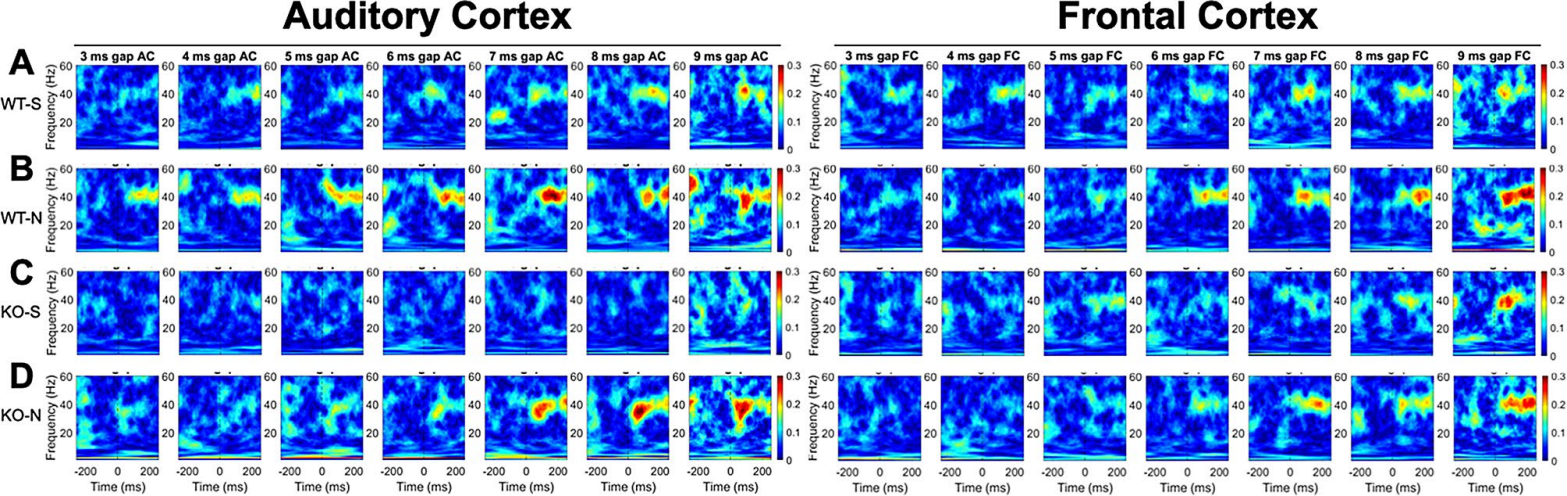
Representative gap-ASSR ITPC heatmaps from auditory and frontal cortex of *Fmr1* WT and KO mice at P21. Each panel shows the ITPC (scale is seen at the right edge of the last panel in AC and FC data, warmer colors mean greater ITPC) obtained from individual animals at a specific gap width. Each column shows ITPC for the same gap width, with the gap width increasing from left to right. As expected, ITPC increases with increasing gap width. The y-axis of each panel is the range of frequencies analyzed for ITPC. Not surprisingly, ITPC is maximum around 40 Hz, which was the frequency of gap-ASSR stimulus. The data shown for AC and FC in each row is from the same mice, with different example mice shown in the different rows. **A**) WT saline. **B**) WT NLX-101. **C**) KO saline. **D**) KO NLX-101. In these examples, mice treated with NLX-101 show higher ITPCs

**Fig. 6:**
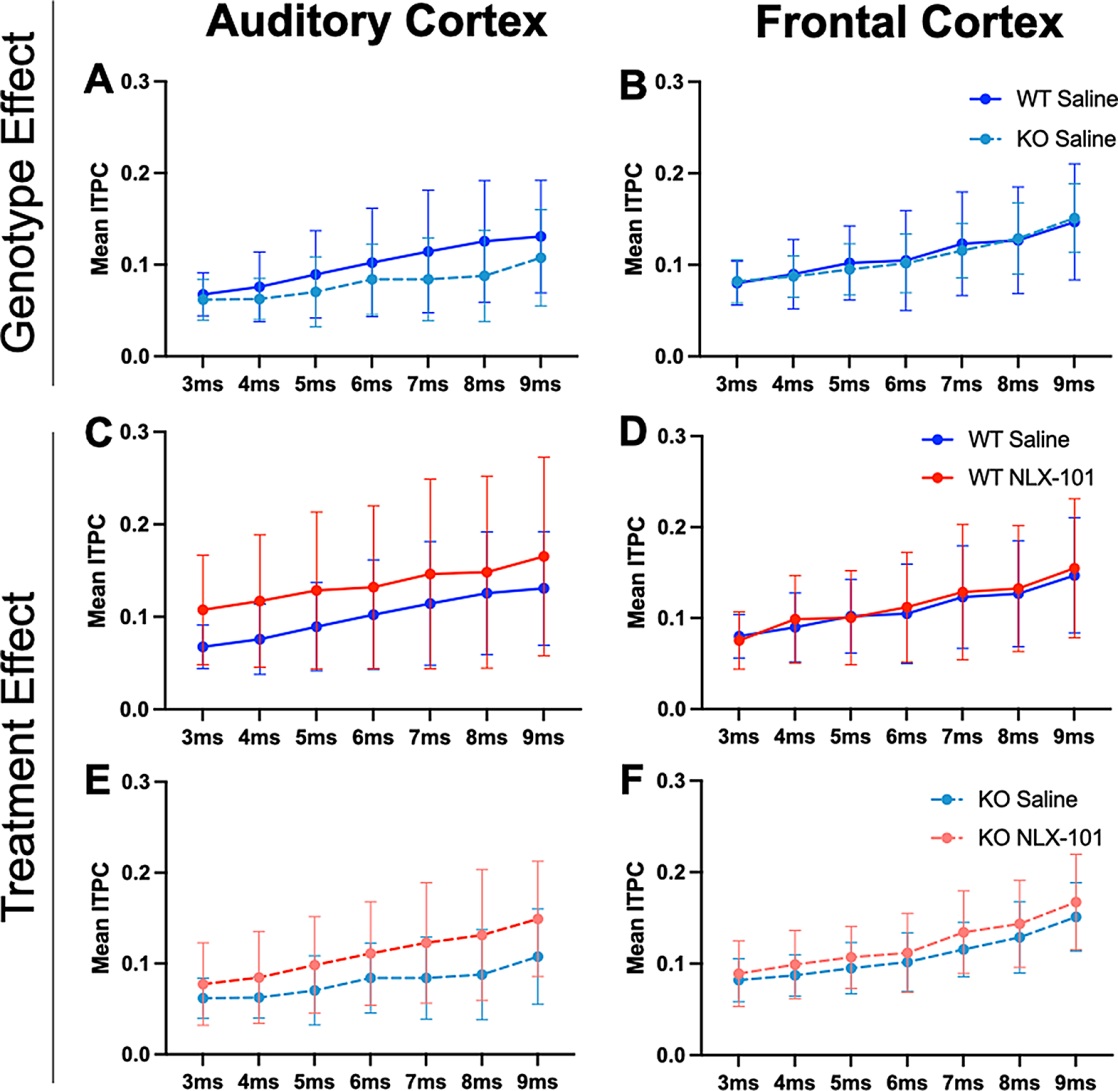
No genotype difference in ITPC was found at P21, but NLX-101 treatment increased ITPC in KO mice in the auditory cortex. **A-B**) No significant difference in ITPC for gap-ASSR was seen between *Fmr1* WT and KO mice in the auditory and frontal cortex. **C-F**) Treatment effect of NLX-101 was found significant with three-way ANOVA. NLX-101 significantly increased ITPC in the auditory, but not the frontal, cortex. Full statistics report is in Table 4. *p < 0.05, **p < 0.01, ***p < 0.001, ****p < 0.0001. Error bars show standard deviation

Figure 5 shows gap-ASSR heat maps of ITPC from both AC and FC of representative mice under each condition at P21: saline treated WT (Fig. 5A), NLX-101 treated WT (Fig. 5B), saline treated KO (Fig. 5C), and NLX-101 treated KO (Fig. 5D). Each panel shows the ITPC at a specific gap width in a single mouse. The columns (left to right) show increasing gap widths. Warmer colors indicate higher ITPC, with the ITPC scale shown at the right end of each row of heat maps. Sound onset is at 0 msec. In these examples, ITPC is highest at 40 Hz as expected, given that the gap stimulus was inserted at 40 Hz in the background noise. Also as expected, ITPC improved with gap width. These illustrative examples suggest that NLX-101 treatment improved ITPC. The population data quantification (Fig. 6) supports these suggestions. To statistically evaluate genotype and treatment effects, repeated three-way ANOVA was adopted (see Method for details). But the effects of genotype and treatment are presented in figures separately for visual clarity. Figure 6A-B shows averaged ITPC in the AC and FC across the gap widths of 3-9ms in saline treated P21 *Fmr1* WT and KO mice. No genotype effect was found in either AC (Repeated three-way ANOVA. *p* = 0.0553) or FC (Repeated three-way ANOVA. *p* = 0.9706). Comparison of averaged ITPC between saline and NLX-101 treatment in the AC (Fig. 6C and E) and FC (Fig. 6D and F) revealed that acute NLX-101 administration significantly increased ITPC in the AC, but not the FC (Fig. 6C-F Repeated three-way ANOVA. AC: *p* = 0.0257; FC: *p* = 0.5771). Full statistical results of gap-ASSR at P21 are shown in Table 4. Therefore, NLX-101 showed improvement of temporal processing in the AC at P21.

**Table 4:**
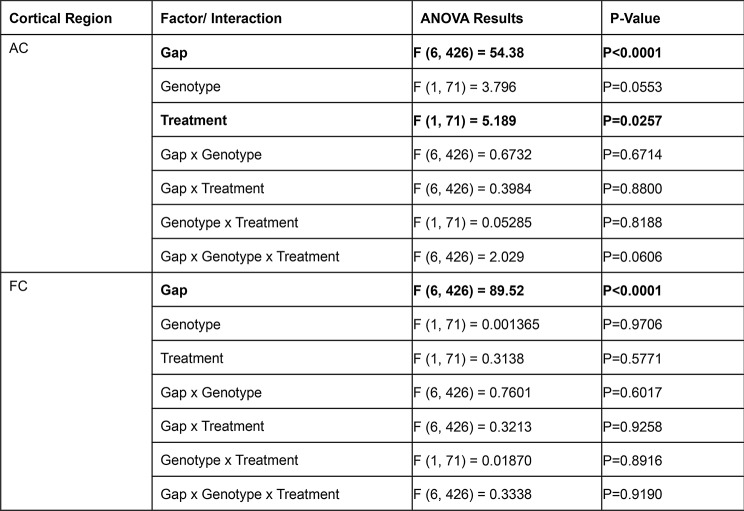
Full statistical analysis at P21 gap-ASSR data

**Fig. 7:**
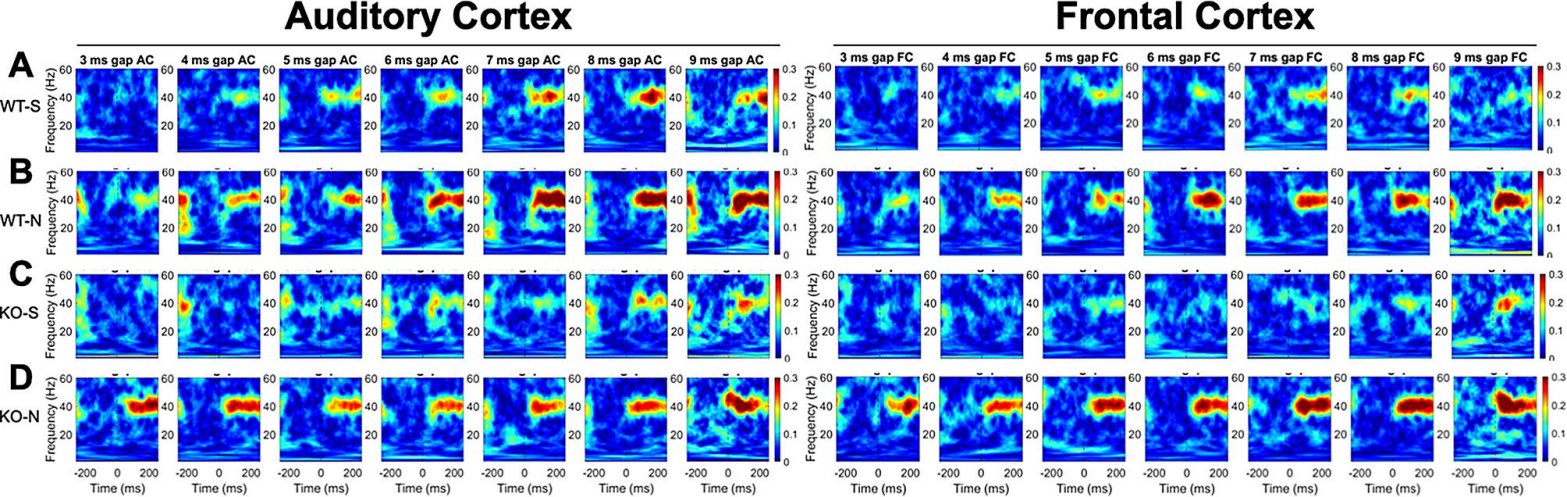
Representative gap-ASSR ITPC heatmaps from auditory and frontal cortex of *Fmr1* WT and KO mice at P30. **A**) WT saline. **B**) WT NLX-101. **C**) KO saline. **D**) KO NLX-101. Details as in Fig. 5

**Fig. 8:**
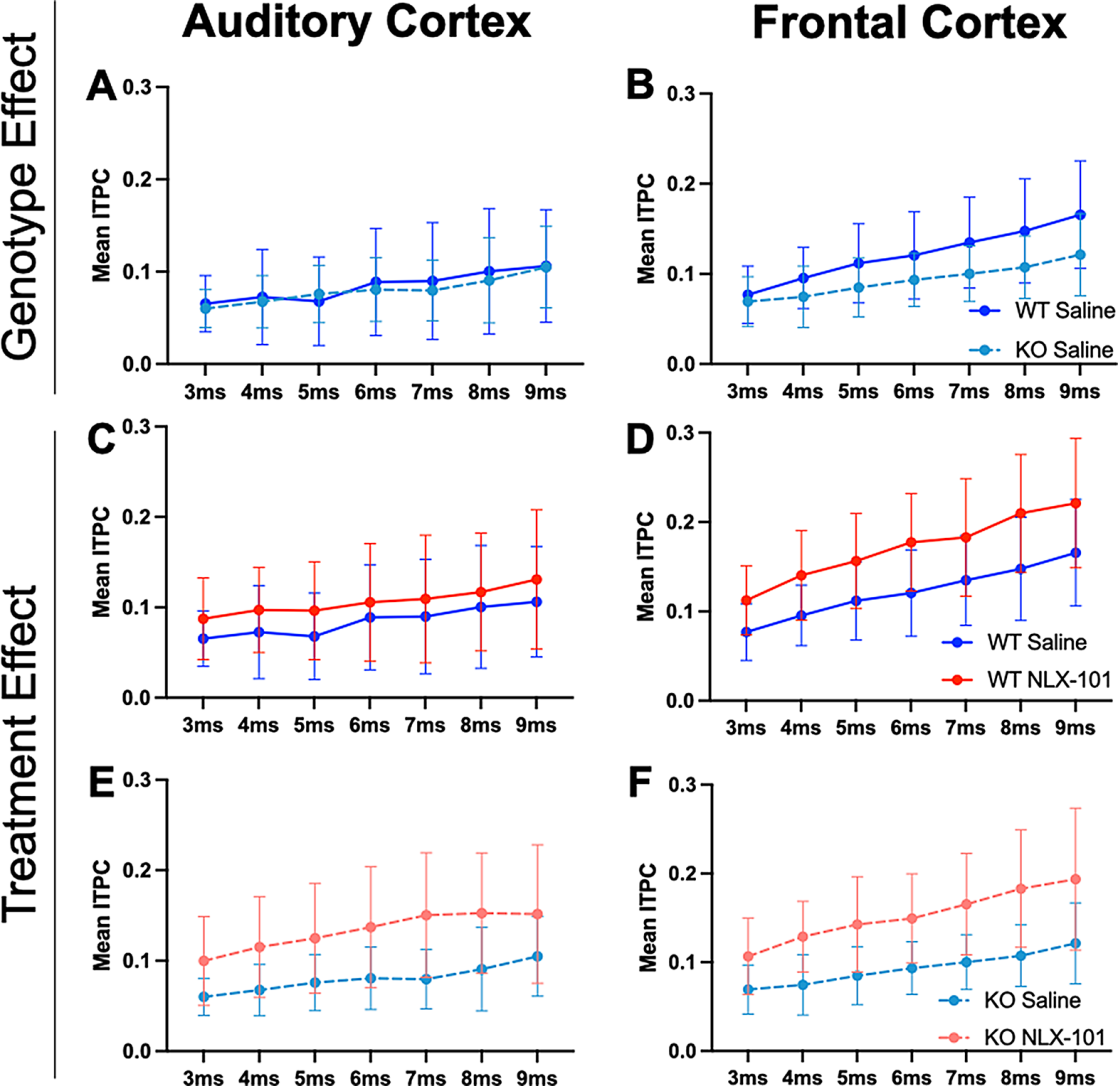
Genotype difference in ITPC was found in frontal, but not auditory, cortex at P30. NLX-101 increased ITPC in *Fmr1* WT and KO mice. **A-B**) No genotype difference in gap-ASSR ITPC was found in the auditory cortex, but ITPC was significantly lower in the frontal cortex of *Fmr1* KO mice compared with WT controls. **C-F**) Acute NLX-101 administration significantly increased ITPC in both auditory and frontal cortex. Besides, NLX-101 increased ITPC as gap width increases in the frontal cortex (gap width x treatment interaction). WT exhibited higher ITPC than KO at longer gap widths (gap width x genotype interaction). Full statistics report is in Table 5. *p < 0.05, **p < 0.01, ***p < 0.001, ****p < 0.0001. Error bars show standard deviation

Figure 7 shows gap-ASSR heat maps of ITPC from both AC and FC of representative mice under each condition at P30: saline treated WT (Fig. 7A), NLX-101 treated WT (Fig. 7B), saline treated KO (Fig. 7C), and NLX-101 treated KO (Fig. 7D). NLX-101 improves ITPC in these mice, compared to saline. Across the population of mice, at P30, ITPC during gap-ASSR stimuli was significantly reduced in *Fmr1* KO mice compared to WT controls in the FC (Fig. 8B, repeated three-way ANOVA. *p* = 0.0184), but not in the AC (Fig. 8A, repeated three-way ANOVA. *p* = 0.3130). Acute NLX-101 administration significantly increased ITPC in both the AC and the FC (Fig. 8C-F Repeated three-way ANOVA. AC: *p* = 0.0010; FC: *p* < 0.0001), indicating broad benefits to temporal processing in developing mice. Besides, we found ITPC in WT is significantly higher than that in KO at longer gap widths in the FC but not the AC (Three-way ANOVA. Gap x Genotype, FC: *p* = 0.0021; AC: *p* = 0.5647). Compared with shorter gap widths, NLX-101 exhibited greater effect at longer gap withs in the FC (Three-way ANOVA. Gap width x Treatment: *p* = 0.0012), but not in the AC (Three-way ANOVA. Gap width x Treatment: *p* = 0.7439). Full statistical results of NLX-101 on gap-ASSR at P30 are shown in Table 5. Taken together, significant deficits in temporal processing were seen in saline treated *Fmr1* KO mice at P30. Temporal processing at P30 was improved in both cortical regions in the *Fmr1* KO mice with NLX-101.

**Table 5:**
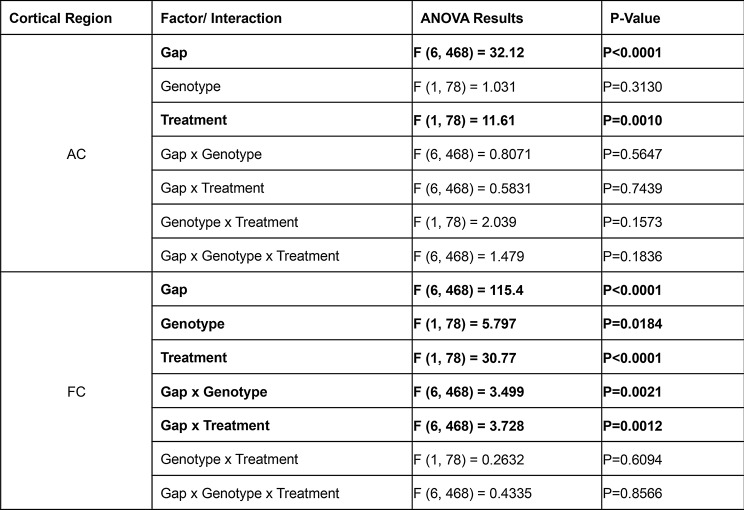
Full statistical analysis at P30 gap-ASSR data

### Sex difference in temporal processing was observed in WT group

Sex differences were examined in all measures, but significant results were only seen in ITPC. Sex difference in temporal processing was only found in WT group at both P21 (Figure S1) and P30 (Figure S2), but not in the KO group at either age (Figure S3 and Figure S4). At P21, saline treated WT females showed significantly higher ITPC in the FC than the male counterpart (Figure S1 B, repeated two-way ANOVA. Sex effect: *p* = 0.0266). At P21, the NLX-101 treated WT females exhibited higher ITPC at longer gap width than the male counterpart (Figure S1 D, repeated two-way ANOVA. Gap width × Sex: *p* = 0.0249). Full statistical results of sex differences of WT at P21 in gap-ASSR are shown in Supplementary Table S1 At P30, sex difference in ITPC was only found in the treatment group where NLX-101 treated males had higher ITPC than female counterpart (Figure S2 D, repeated two-way ANOVA. *p* = 0.0299). Full statistical results of sex differences of WT at P30 in gap-ASSR are listed in Supplementary Table S1 Full statistical results of sex differences of KO at P21 and P30 in gap-ASSR can be found in Supplementary Tables S3 and S4 respectively. Taken together, the effect of NLX-101 in improving temporal processing is not specific to FXS, instead, there might be a shared underlying circuit of temporal processing under serotonin modulation. Such modulation may differ in male and female at early age point (P21).

## Discussion

This study investigated the effects of acute injection of NLX-101, a selective serotonin-1 A receptor biased agonist, on EEG phenotypes in *Fmr1* KO mice. Consistent with previously published studies (reviewed in [32], EEG phenotypes in *Fmr1* KO mice including elevated ERP amplitudes, enhanced STP and reduced ITPC were found in this study in saline-treated *Fmr1* KO mice. These data add support to the robust and replicable nature of the EEG phenotypes in *Fmr1* KO mice across several studies and mouse strains (Increased ERP amplitudes [29, 30, 56], Increased STP [29, 30], Decreased ITPC [30, 56, 58]). These phenotypes are also translationally relevant, given the similarity across mice and humans, and should be included in the battery of pre-clinical tests for therapeutic development. Following injection of NLX-101, ITPC in both *Fmr1* WT and KO was improved in both P21 and P30 groups; STP in *Fmr1* KO group was significantly reduced to the level that was indistinguishable to the WT control at P30, but not at P21; ERP in both age groups were not affected by the treatment. These data show that the EEG phenotypes are present from early development. NLX-101 shows robust engagement with EEG measures with specificity to alter STP and gap-ASSR measures, without affecting ERP amplitudes. Taken together, these data suggest a promising clinical treatment pathway in FXS by targeting 5-HT_1A_ receptors in early development either alone, or in combination with other treatments that may reduce ERP amplitudes.

### Hypersensitivity in *Fmr1* KO mice

Broadband noise was used to evoke auditory ERP responses in the present study. Background non-phase locked brain activity during noise stimulation was measured as single trial power (STP). In humans, the ERP N100 (N1 in mice) is tied closely to sound detection and arises from activity in the primary and secondary auditory cortices [59, 60]. ERP N1 amplitudes reflect cortical processing with increased amplitudes occurring due to increased neural responses and/or increased synchrony of responses in the population [61]. In the auditory cortex, inferior colliculus and superior olive of *Fmr1* KO mice, there is elevated sound driven neural activity and background activity [14, 36, 37, 62]. Indeed, enhanced evoked responses have been reported in other sensory cortices as well. In primary somatosensory cortex (S1), tactile stimulation of the hind paw produced significantly higher EPSP amplitudes and spiking rates in *Fmr1* KO mice than in WT mice [63]. Increased synchrony of neurons is also seen in sensory cortex of *Fmr1* KO mice [64]. Together, increased sound-driven spiking response and synchrony may explain increased N1 amplitude in FXS.

The increased gamma band STP in *Fmr1* KO mice reflect elevated non-phase locked power during acoustic stimulus presentation and is the summed single trial power both during and in-between stimuli. The gamma band STP change was not limited to low (40–60 Hz) or high gamma (60–100 Hz) frequencies, but encompassed both, and may reflect enhanced background cortical activity [65] and reduced activation of parvalbumin expressing cortical GABAergic neurons in the *Fmr1* KO mice [66]. These STP data during development of *Fmr1* KO mice are consistent with adult mice [30] which also show increased gamma (but not lower) band STP. This STP result is also consistent with studies across rodents that examined resting state (no stimulus) EEG power and showed elevation in gamma band, both low and high [30, 67]. We did not observe differences in STP at lower frequencies [9, 10]. Studies in humans with FXS also shows increased gamma STP during acoustic stimulation. However, it is unclear if lower frequency STP changes are seen as different outcomes have been reported in two different studies [9, 10]. Studies in humans with FXS and rodent models also show elevated resting state EEG gamma power. Whether the elevated gamma STP is simply a reflection of baseline condition, or if background is further elevated during acoustic stimulation cannot be disambiguated from our data. Regardless, elevated background cortical activity in humans and mice will reduce signal to noise ratio during acoustic processing. In humans with FXS, elevated STP is correlated with distractibility and communication measures, suggesting potential clinical implications [10]. Increased background activity has been observed in *Fmr1* KO mice in other sensory cortices as well. In S1, the percentage of neurons firing action potentials spontaneously is significantly higher in *Fmr1* KO mice than in WT mice [63]. Visual cortex in *Fmr1* KO rats was found to maintain “active” states even in the absence of arousal and attention [68]. Traditionally, background neural activity is viewed as “noise”, but spontaneous cortical activity interacts with external stimulation to produce behavioral responses toward sensory stimuli [69, 70]. Given that cortical responses are shaped by both external stimuli and spontaneous activity [69], elevated evoked ERP amplitudes seen in *Fmr1* KO group in this study may also be partially explained by the increased STP in *Fmr1* KO mice. However, the differential impact of NLX-101 on ERP amplitude versus STP suggests they are generated by relatively independent mechanisms, most likely elevated synchrony playing a role in N1 amplitudes, and elevated responses playing a role in STP.

### Effect of NLX-101 in reducing auditory hypersensitivity

Single unit recordings in the inferior colliculus suggest how NLX-101 may reduce STP in *Fmr1* KO mice [71]. The target of NLX-101, 5-HT_1A_ receptors, are the predominant inhibitory 5-HT receptor subtype, decreasing cAMP production via activation of Gα_i_ proteins [72], a process that is dysregulated in FXS [73]. Consistent with the hyperpolarizing effect of 5-HT_1A_ activation, in the inferior colliculus, activation of 5-HT_1A_ receptors with 8-OH-DPAT narrowed the response window of individual neurons by suppressing the latter spikes in response to sounds [71, 74]. Indeed, *Fmr1* KO mouse single unit recordings show that ongoing responses after the stimulus, but not onset responses, are elevated in the KO mice compared to WT mice [36]. Besides, neurons with longer first-spike latencies have a higher tendency to be suppressed by 8-OH-DPAT than those with shorter first-spike latencies [71]. By narrowing the response window, and by reducing overall spiking in the midbrain, NLX-101 may reduce STP recorded in the auditory cortex. However, the drug appears to have minimal effect on cortical synchrony, leaving ERP amplitudes unchanged. The notion that NLX-101 has main effect in the midbrain is also supported by the data that showed the drug essentially abolished audiogenic seizures, a phenotype that potentially originates in the midbrain of *Fmr1* KO mice [75]. The improved effect of the drug on STP at P30, compared with P21, may reflect developmental regulation of 5-HT_1A_ receptors and/or reduction of neural activity in the midbrain between P21 and P30. Future studies should examine P21 STP with a higher acute dose of NLX-101.

### Increased trial-to-trial variability in FXS

Consistent behavior output largely relies on reliable sensory perception. In speech comprehension, for example, variable auditory processing will lead to unstable perception and is likely to underlie speech and language differences in FXS and other sensory-related cognition measures [76]. Varied behavioral responses in autism have been broadly reported in human studies including significantly increased trial-by-trial variability in multiple sensory modalities (Auditory: [77, 78]. Visual: [77, 79, 80]. Somatosensory: [77, 81]). In this study, we examined temporal fidelity of cortical responses in *Fmr1* KO and WT mice with a gap-in-noise auditory-steady-state response (ASSR) paradigm. ASSR measures the capability of the auditory system to accurately phase lock to temporally modulated sound stimuli [82]. A previous study suggested trial-by-trial variability tends to be higher when sensory stimulus is more complex [76]. Therefore, instead of using 40-Hz click-train stimulus to induce 40 Hz auditory oscillations in the conventional ASSR [83], we inserted gaps at 40 Hz in the continuous background noise to make the stimuli more challenging to synchronize with (at short gaps, in particular), to better assess temporal processing acuity in mice [40]. The consistency of auditory responses can be measured using the inter-trial phase clustering (ITPC) which quantifies phase locking fidelity across trials. In line with published studies that used different spectrotemporally modulated stimuli [30, 56, 58], reduced ITPC was observed in *Fmr1* KO group compared with the WT group. Such reduced ITPC, or increased variability from trial to trial, at the neural network level, may be related to variability at the cellular level such as variable resting membrane potentials of individual neurons in the *Fmr1* KO mice [63]. Cortical recordings to sounds also showed increased variability across trials in terms of latency [14], which will lead to variable representation of temporal responses. It should be noted that a previous study [27]examined genotype differences between WT and *Fmr1* KO mice n 40 Hz gap-ASSR ITPC. Consistent with the current study, reduced ITPC was seen in the FC of *Fmr1* KO mice at P30 and no deficits were present in the AC. However, in contrast to the present study, reduced FC ITPC was reported at P21 as well in the KO mice. The reasons for these differences are unclear, but may be related to the fact that all mice in the present study were injected (saline or NLX-101) before EEG recordings. It is possible the mild stress of handling and injection disrupts behavior at P21, but not at P30.

Our data suggest NLX-101 functions to reduce trial-to-trial variability as measured by increased ITPC. However, it is not known if abnormal 5-HT_1A_ receptor function underlies increased trial-to-trial variability in the *Fmr1* KO mice. Both WT and KO mice, and at both ages tested, showed improved ITPC with NLX-101, suggesting serotonin modulation reduces trial-by-trial variability. Serotonin and 5-HT_1A_ agonists (8–0 H-DPAT) reduce response gain in sensory responses [71, 84], which could potentially lead to less trial-to-trial variability. In the inferior colliculus, 5-HT_1A_ receptor activation reduces response duration, which could also reduce response variability.

Our data on the utility of modulating serotonin signaling in *Fmr1* KO mice is consistent with the notion that the serotonin system may provide potentially useful therapeutic pathways to treat FXS (reviewed in [85]). The serotonin transporter (5-HTTLPR) genotype correlated with more aggressive, destructive, and stereotypic behaviors in humans with FXS (ages 8–24 years) [86]. A recent study revealed that juvenile *Fmr1* KO mice had lower whole-brain 5-HT_1A_ receptor expression than WT mice [33]. Costa et al., (2012,2018) [87, 88] found that stimulation of 5-HT_7A_ receptors reversed the consistently exaggerated hippocampal mGluR5-mediated synaptic plasticity defects in *Fmr1* KO mice to WT range, and improved learning outcomes. Lim et al., (2014) [89] showed that psychoactive drugs that act on 5-HT and dopamine receptors improved learning in Y-maze and fear-conditioning paradigms in the *Fmr1* KO mice. Importantly they suggested low-dose activation of both receptor types to be beneficial, setting the stage to examine if NLX-101 in combination with other drugs may reduce most, if not all, of the EEG phenotypes. Saraf et al., (2022) [90] found that FPT, a non-selective agonist of several 5-HT_1_ and 5-HT_7A_ receptors engaged spectral band changes EEG recordings from *Fmr1* KO mice (alpha and delta power changes, but not in the gamma band), reduced audiogenic seizures and improved social behaviors [17, 33]. Fluoxetine, a selective serotonin reuptake inhibitor (SSRI), has some anxiolytic effect in *Fmr*1 KO mice, and reduces hyperactivity. Developmental changes in the serotonin transporter and BDNF/ TrkB signaling may underlie some differences in effects in WT versus *Fmr1* KO mice [91]. Sertraline, another SSRI, also shows off-label efficacy to improve language function in FXS [92]. We observed a more prominent effect of NLX-101 at P30 compared to P21 (STP and gap-ASSR) in the *Fmr1* KO mice. This also may be related to developmental changes in 5-HT_1A_ receptors. Indeed, our unpublished work shows that the level of 5-HT_1A_ receptor mRNA in the auditory cortex is significantly higher at P30 than that at P21, suggesting more 5-HT_1A_ receptors are available at P30 for NLX-101 modulation. The more specific effect of NLX-101 on the *Fmr1* KO, but not WT, mice (e.g., with STP at P30) has been observed with other pharmacological treatments. For example, the BK channel modifier Chlorzoxazone has specific effects on behavior and neural activity in adult *Fmr1* KO mice, but not in WT mice [93]. In these treatments, it is possible that the drug only acts to modify the pathological state, and does not affect sensitivity of channels or receptors under normal activation levels.

### Future directions

Several future studies are suggested by our results. NLX-101 did not induce tachyphylaxis in the AGS study [22]. Future studies should evaluate effects following chronic dosing to determine if there is any tachyphylaxis in terms of EEG outcomes. Here, NLX-101 was applied systemically, so it is unclear whether the observed improvement is contributed predominantly by one or more brain regions, although previous studies on NLX-101 indicate that it preferentially targets 5-HT_1A_ receptors in cortical and brainstem regions. Future studies that specifically administer 5-HT_1A_ receptor agonists and/ or antagonists in the inferior colliculus or auditory cortex will identify regional effects. The present study was done with an acute single injection of NLX-101, and studies are warranted to determine whether its effects are maintained upon chronic administration. The expression levels of 5-HT_1A_ receptors may change with age, leading to specific optimal treatment windows, so future studies will examine expression of 5-HT_1A_ receptors across development, regions, and age. Cortical hyperexcitability is present in *Fmr1* KO mice and human with FXS. It is likely that such hyperexcitability drives behavioral sensory hypersensitivity and abnormal temporal processing. In humans with FXS, there are clinical correlations with EEG measures suggesting a behavioral significance. Future studies should develop paradigms to test the causal relationship between EEG outcomes and behavioral phenotypes.

## Conclusions

Our findings suggest 5-HT_1A_ receptor modulation as a useful therapeutic approach in FXS. NLX-101 has specific properties that may be of use in treatment of sensory variability and background noise in FXS. Unlike NLX-101, the more commonly used agonist of 5-HT_1A_ receptors, 8-OH-DPAT, also activates autoreceptors in the raphe nuclei [94] and causes hypothermia [95, 96]. In contrast, NLX-101 has much higher selectivity compared with 8-OH-DPAT [25] and preferentially activates 5-HT_1A_ heteroreceptors [24], leaving autoreceptors in the raphe nuclei unaffected. The unique neuropharmacology of such biased agonists may make them more suitable for therapeutic approaches, and future studies should determine if NLX-101 or other biased selective 5-HT_1A_ receptor agonists are beneficial in children with FXS.

## Electronic supplementary material

Below is the link to the electronic supplementary material.

Supplementary Material 1

Supplementary Material 2

## Data Availability

Availability of Data and Materials: The data that support the findings of this study are available from the corresponding author with reasonable request.

## Abbreviations

ASD: Autism spectrum disorders
AGS: Audiogenic seizures
KO: Knock-out
FXS: Fragile X syndrome
WT: Wild-type
ERP: Event-related potentials
ASSR: Auditory steady-state response
P: Postnatal
Fmr1: Fragile X messenger ribonucleoprotein gene
FMRP: Fragile X messenger ribonucleoprotein
AC: Auditory cortex
FC: Frontal cortex
i.p.: Intraperitoneal
STP: Single trial power
ITPC: Inter-trial phase clustering
