# Supplementary material for: Acute administration of NLX-101, a Serotonin 1A receptor agonist, improves auditory temporal processing during development in a mouse model of Fragile X Syndrome
Source: J Neurodev Disord. 2025 Jan 3;17:1. doi: 10.1186/s11689-024-09587-0 (PMC11697955; doi:10.1186/s11689-024-09587-0)
Supplement: Supplementary file 1 — Supplementary Material 1 [file 11689_2024_9587_MOESM1_ESM.pdf]

Additional File 5. (Supplementary Table 1) Full statistical analysis of sex differences of WT at P21 gap-ASSR data

Group	Cortical Region	Factor/ Interaction	ANOVA Results	P Value
WT_Saline_P21	Auditory Cortex	Gap width x Sex	F (6, 102) = 0.2516	0.9576
		**Gap Width**	**F (2.084, 35.44) = 16.06**	**<0.0001**
		Sex	F (1, 17) = 0.2682	0.6112
	Frontal Cortex	**Gap width x Sex**	**F (6, 102) = 4.321**	**0.0006**
		**Gap Width**	**F (3.476, 59.08) = 23.92**	**<0.0001**
		**Sex**	**F (1, 17) = 5.892**	**0.0266**
WT_NLX-101_P21	Auditory Cortex	Gap width x Sex	F (6, 108) = 0.08309	0.9978
		**Gap Width**	**F (2.582, 46.47) = 10.84**	**<0.0001**
		Sex	F (1, 18) = 0.001554	0.9690
	Frontal Cortex	**Gap width x Sex**	**F (6, 108) = 2.530**	**0.0249**
		**Gap Width**	**F (3.153, 56.76) = 22.31**	**<0.0001**
		Sex	F (1, 18) = 0.4625	0.5051

Two-way repeated ANOVA results for gap-ASSR WT data analysis at P21. Geisser-Greenhouse correction was applied for sphericity when necessary. P-vale was corrected for multiple comparisons using Sidak method. Bold text indicates statistical significance (p ≤ 0.05).

Additional File 6. (Supplementary Table 2) Full statistical analysis of sex differences of WT at P30 gap-ASSR data

Group	Cortical Region	Factor/ Interaction	ANOVA Results	P Value
WT_Saline_P30	Auditory Cortex	Gap width x Sex	F (6, 108) = 0.5677	0.7552
		**Gap Width**	**F (3.231, 58.16) = 7.810**	**<0.0001**
		Sex	F (1, 18) = 0.6768	0.4215
	Frontal Cortex	Gap width x Sex	F (6, 108) = 1.172	0.3269
		**Gap Width**	**F (3.892, 70.05) = 28.30**	**<0.0001**
		Sex	F (1, 18) = 0.8938	0.3570
WT_NLX-101_P30	Auditory Cortex	Gap width x Sex	F (6, 108) = 0.2326	0.9651
		**Gap Width**	**F (2.550, 45.91) = 5.232**	**0.0052**
		Sex	F (1, 18) = 0.01807	0.8946
	Frontal Cortex	Gap width x Sex	F (6, 108) = 0.5645	0.7577
		**Gap Width**	**F (3.487, 62.76) = 49.25**	**<0.0001**
		**Sex**	**F (1, 18) = 5.557**	**0.0299**

Two-way repeated ANOVA results for gap-ASSR WT data analysis at P30. Geisser-Greenhouse correction was applied for sphericity when necessary. P-vale was corrected for multiple comparisons using Sidak method. Bold text indicates statistical significance ($p \leq 0.05$).

Additional File 7 (Supplementary Table 3) Full statistical analysis of sex differences of KO at P21 gap-ASSR data

Group	Cortical Region	Factor/ Interaction	ANOVA Results	P Value
KO_Saline_P21	Auditory Cortex	Gap width x Sex	F (6, 114) = 0.8582	0.5281
		**Gap Width**	**F (2.682, 50.95) = 16.06**	**<0.0001**
		Sex	F (1, 19) = 0.01326	0.9095
	Frontal Cortex	Gap width x Sex	F (6, 114) = 0.7417	0.6171
		**Gap Width**	**F (4.397, 83.58) = 32.47**	**<0.0001**
		Sex	F (1, 19) = 0.1451	0.7075
KO_NLX-101_P21	Auditory Cortex	Gap width x Sex	F (6, 114) = 0.5142	0.7966
		**Gap Width**	**F (2.354, 44.72) = 23.28**	**<0.0001**
		Sex	F (1, 19) = 0.01842	0.8935
	Frontal Cortex	Gap width x Sex	F (6, 114) = 0.3490	0.9092
		**Gap Width**	**F (3.508, 66.66) = 35.58**	**<0.0001**
		Sex	F (1, 19) = 0.02423	0.8779

Two-way repeated ANOVA results for gap-ASSR KO data analysis at P21. Geisser-Greenhouse correction was applied for sphericity when necessary. P-vale was corrected for multiple comparisons using Sidak method. Bold text indicates statistical significance ($p \leq 0.05$).

Additional File 8. (Supplementary Table 4) Full statistical analysis of sex differences of KO at P30 gap-ASSR data

Group	Cortical Region	Factor/ Interaction	ANOVA Results	P Value
KO_Saline_P30	Auditory Cortex	Gap width x Sex	F (6, 114) = 2.090	0.0597
		**Gap Width**	**F (3.698, 70.26) = 10.07**	**<0.0001**
		Sex	F (1, 19) = 0.3268	0.5742
	Frontal Cortex	Gap width x Sex	F (6, 114) = 2.145	0.0535
		**Gap Width**	**F (3.579, 68.01) = 15.57**	**<0.0001**
		Sex	F (1, 19) = 0.007057	0.9339
KO_NLX-101_P30	Auditory Cortex	Gap width x Sex	F (6, 114) = 1.569	0.1625
		**Gap Width**	**F (3.132, 59.51) = 11.62**	**<0.0001**
		Sex	F (1, 19) = 0.9161	0.3505
	Frontal Cortex	Gap width x Sex	F (6, 114) = 0.2151	0.9714
		**Gap Width**	**F (2.373, 45.09) = 25.09**	**<0.0001**
		Sex	F (1, 19) = 0.9769	0.3354

Two-way repeated ANOVA results for gap-ASSR KO data analysis at P30. Geisser-Greenhouse correction was applied for sphericity when necessary. P-vale was corrected for multiple comparisons using Sidak method. Bold text indicates statistical significance ($p \leq 0.05$).
