# Supplementary figures and images for: Acute administration of NLX-101, a Serotonin 1A receptor agonist, improves auditory temporal processing during development in a mouse model of Fragile X Syndrome
Source: J Neurodev Disord. 2025 Jan 3;17:1. doi: 10.1186/s11689-024-09587-0 (PMC11697955; doi:10.1186/s11689-024-09587-0)

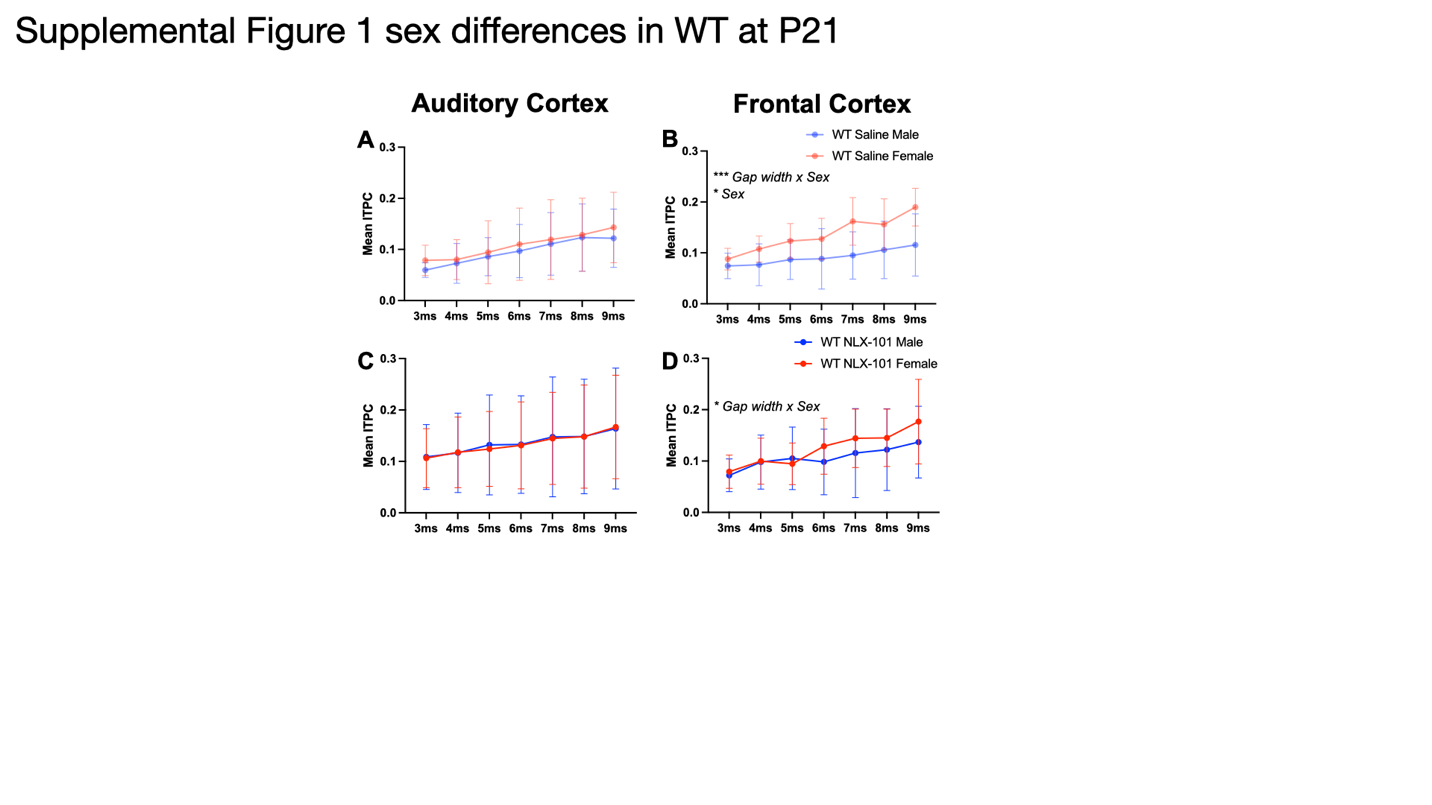


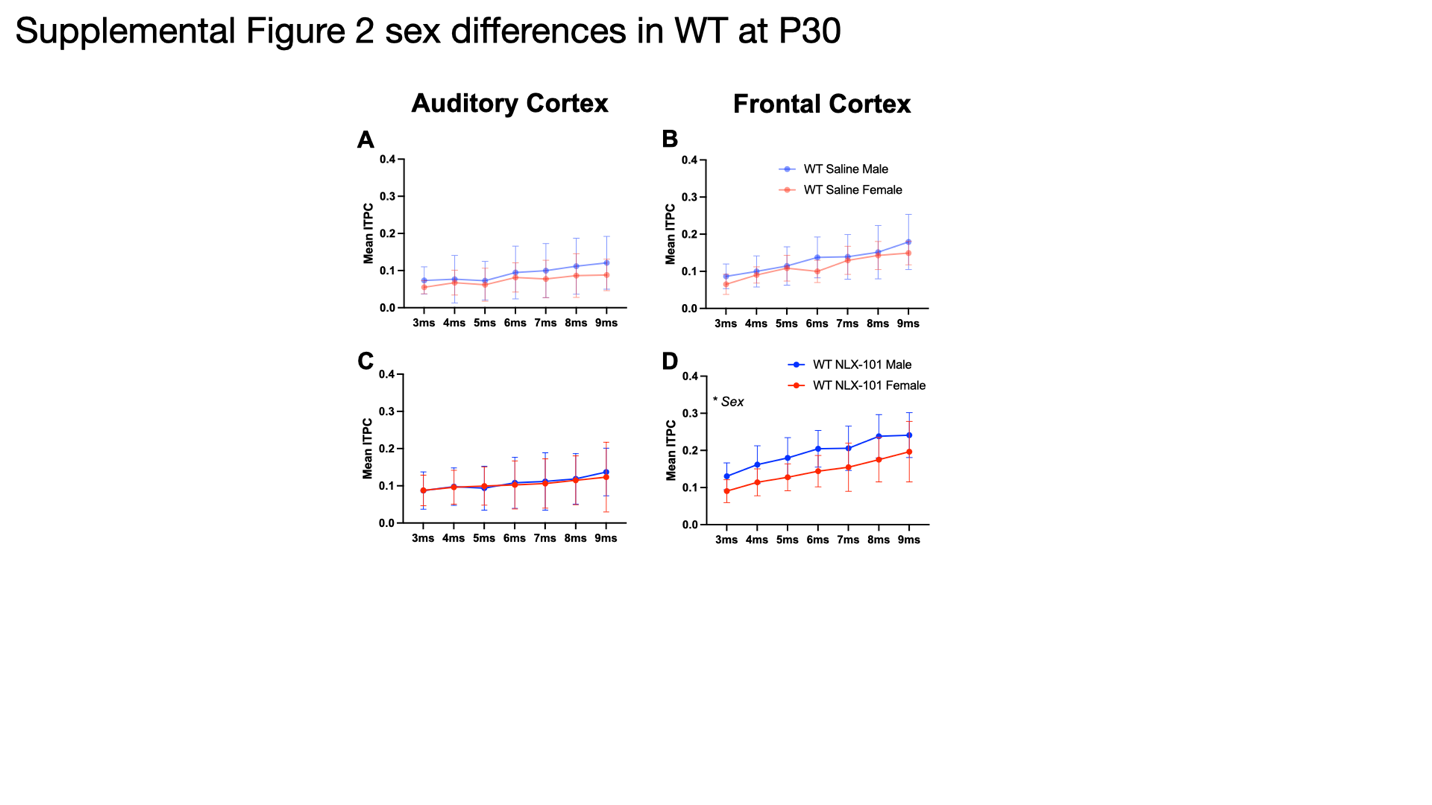


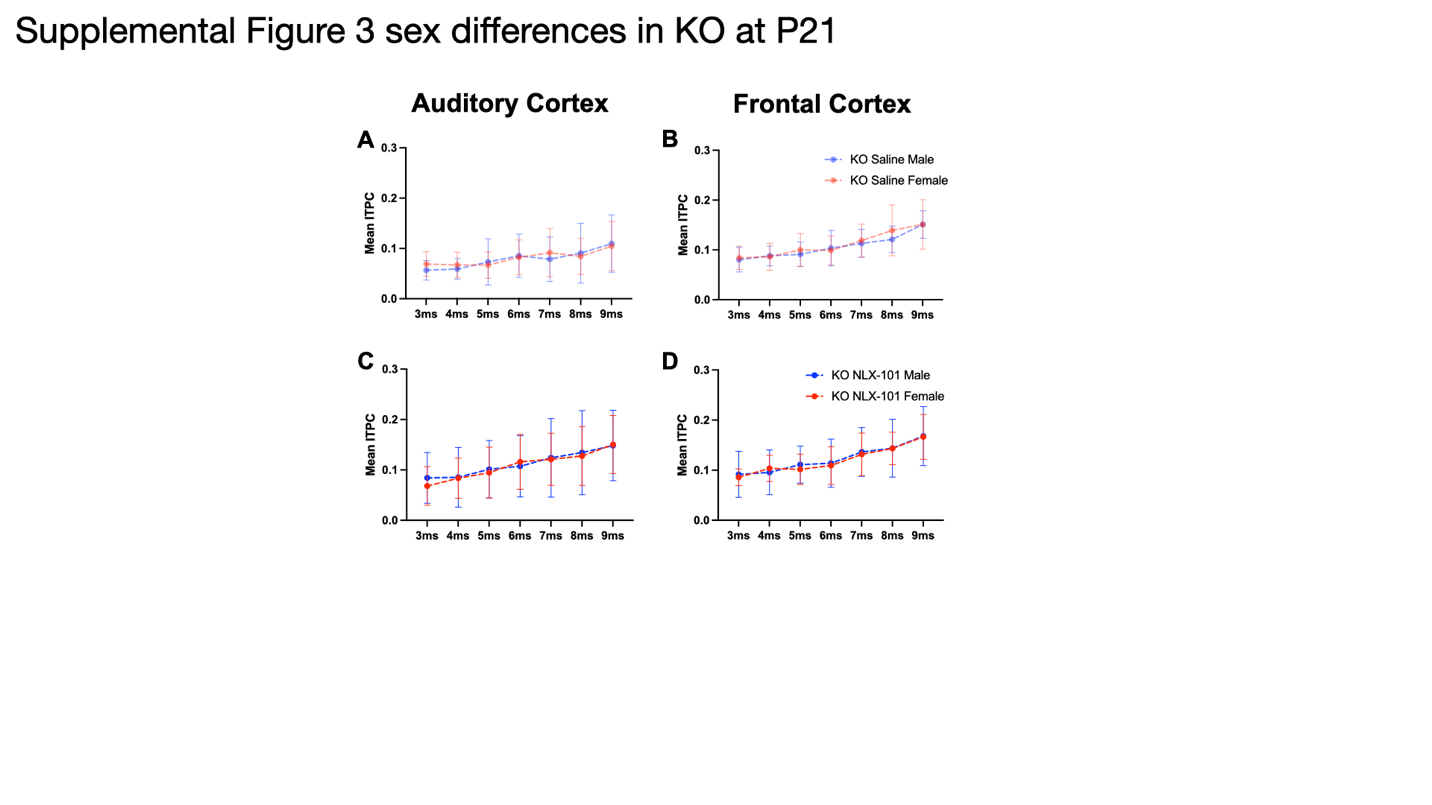


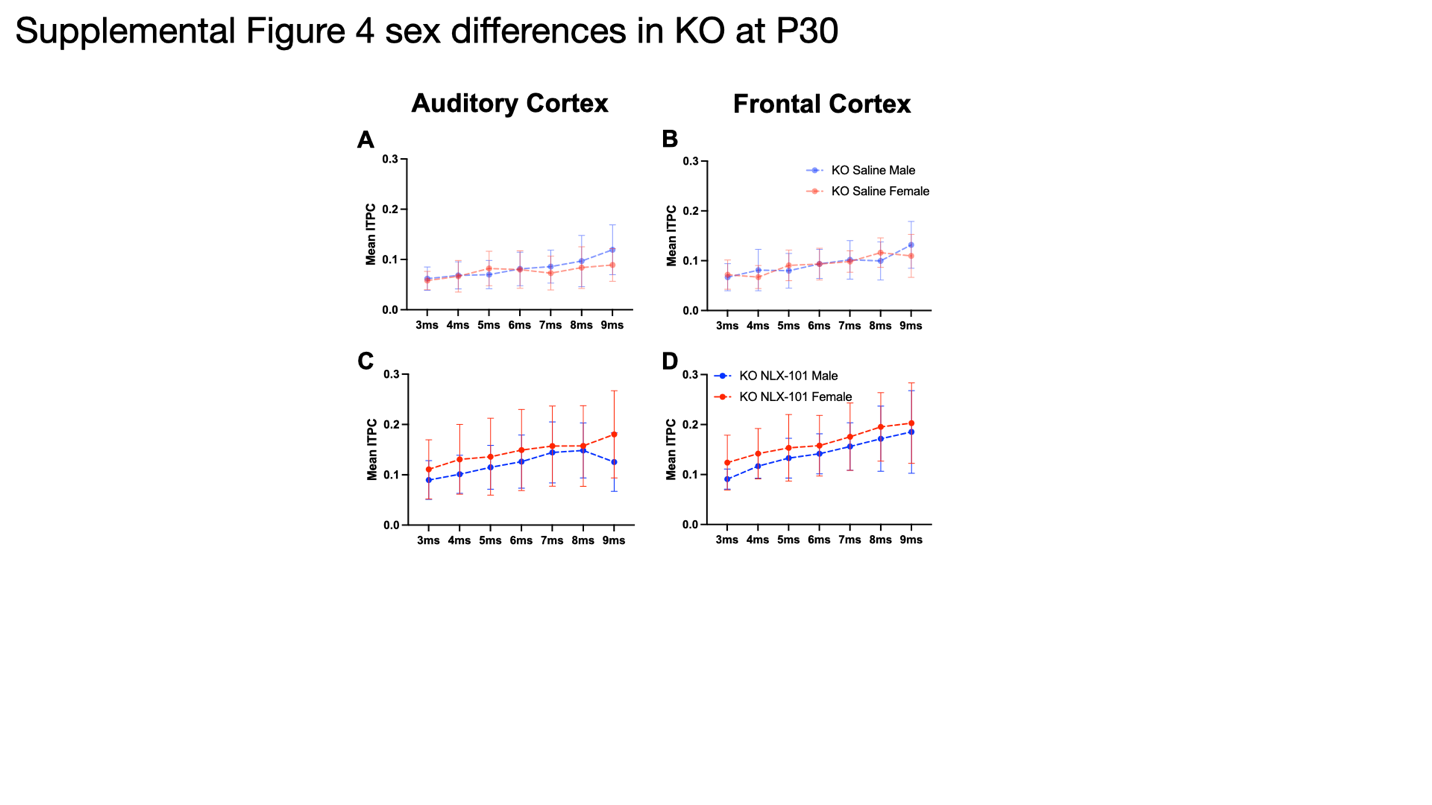

Supplement: Supplementary file 2 — Supplementary Material 2 [file 11689_2024_9587_MOESM2_ESM.docx]
